# Cardiovascular disease prevention practice among adults in Southwest, Ethiopia: a community-based cross-sectional study

**DOI:** 10.11604/pamj.2024.49.112.40080

**Published:** 2024-12-09

**Authors:** Aster Fayisa Dare, Million Abera Berhie, Abebe Abera Tesema, Mamusha Aman Hussen, Bontu Mathewos Getachew, Warkitu Slieshi Ensermu

**Affiliations:** 1 Department of Nursing, Institute of Health, Jimma University, Jimma, Ethiopia,; 2 Departments of Health, Behavior, and Society, Faculty of Public Health, Jimma University, Jimma, Ethiopia,; 3 Department of Nursing, College of Health Science, Dembi Dollo University, Dembi Dolo, Ethiopia,; 4 Department of Midwifery, Institute of Health, Jimma University, Jimma, Ethiopia

**Keywords:** Cardiovascular disease, community, health, prevention, practices

## Abstract

**Introduction:**

recent population-based studies indicated an increasing burden of cardiovascular disease risk factors in sub-Saharan Africa. However, there is limited evidence regarding cardiovascular disease prevention practices among the communities. This study aimed to assess cardiovascular disease prevention practices and associated factors among adults in Jimma Town, Southwest Ethiopia.

**Methods:**

a community-based cross-sectional study was conducted among 634 adults in Jimma Town from August 30^th^ to September 30^th^, 2021. A multistage sampling technique was employed to get the study participants. Data were entered into Epi-data version 4.6 and exported to Statistical Package for Social Science version 23 for analysis. Multivariable logistic regression was conducted to identify potential predictors of cardiovascular disease prevention practice.

**Results:**

the overall good practice was found to be 46.8%. Knowledge of cardiovascular disease risk factors (AOR = 2.013; 95% CI (1.4, 2.9); p < 0.001), self-efficacy (AOR = 1.670; 95% CI (1.1, 2.4); p = 0.007), social support (AOR = 2.063; 95% CI (1.4, 2.9); p < 001), intermediate (AOR = 2.035; 95% CI (1.3, 3.2); p = 0.003) and high (AOR = 2.101; 95% CI (1.3, 3.4); p = 0. 001) self-perceived estimate of cardiovascular disease risk, and working hours (AOR = 0.445; 95% CI (0.3, 0.7); p < 0.001) were significantly associated with cardiovascular diseases prevention practices.

**Conclusion:**

the study found cardiovascular disease prevention practice was suboptimal in the study area. Thus, promoting positive health behaviors regarding cardiovascular disease risk factors in the community is a must.

## Introduction

Cardiovascular diseases (CVD) have continued to be the leading cause of premature death and disability globally for the preceding 20 years. Now, more people are dying from CVD than ever before [1]. According to a World Health Organization (WHO) report in 2019, an estimated 17.9 million people died from CVDs representing 32% of all global deaths [2]. Greater than 80% of CVD deaths take place in Low- and Middle-Income Countries (LMIC) countries and occur almost equally in men and women [3]. While CVD deaths typically occur later in life in high-income countries, in LMIC countries it often affects working-age people, leading to high healthcare costs, limited ability to work, and financial insecurity [4]. Contributing to poverty due to catastrophic health expenditures and extraordinary out-of-pocket expenditures [5,6].

The CVD epidemic in sub-Saharan Africa (SSA) is driven by changes in demographics and altered lifestyles as a result of increasing urbanization and socioeconomic development [6]. With the rising epidemic of CVD in SSA, behavioral modification interventions are essential in supporting populations to attain better cardiovascular health. In addition, inadequate resources and the high cost of CVD treatment necessitate prevention should be a high priority for CVD control in SSA [7,8].

Ethiopia is no exception to the impact of CVD where it is the leading cause of death and a major cause of chronic disease and disability [9]. Now the country is experiencing an epidemiologic change mainly driven by demographic and lifestyle changes. Prompt urbanization has attracted a huge population from rural areas to cities. Urban life endorses a sedentary lifestyle and increases the chance of CVDs. Taking these facts into consideration, the burden of CVDs in Ethiopia might be even higher than anticipated [7,9]. As the study conducted in Jimma Town in 2017 showed the prevalence of CVD risk factors was high wherein 70% of adults were presented with one or more risk factors [10].

However, to the best knowledge of the investigators, there were no studies conducted on factors associated with CVD prevention practices among the community in Ethiopia, particularly in Jimma Town. As a result, this study aimed to identify CVD prevention practices and associated factors among adults in Jimma Town, Southwest Ethiopia.

## Methods

**Study setting and design:** a community-based cross-sectional study was conducted among adults aged 18 years or older in Jimma Town from August 30 to September 30, 2021. Jimma Town is the capital city of Jimma Zone of the Oromia Regional State, located 357 Km away from Addis Ababa, which is the capital city of the country in Southwest Ethiopia [11]. All adults (≥ 18 years) residing in Jimma Town were the source of population and all sampled adult (≥ 18 years) population in Jimma Town who fulfilled the inclusion criteria were the study population. Adults aged 18 years or older who lived in Jimma Town for at least six months and were willing to participate were included in this study. Patients with major psychiatric problems and critical illness that can interfere with the recognition of CVD prevention practices were excluded from the study.

The sample size was determined using the formula for estimating sample size for a single population proportion. Since, no previous similar studies the p-value was taken as 0.5, the margin of error 0.05, and a non-response rate of 10% were used. After a design effect of 1.5, inferred from the WHO estimate for CVD risk factor assessment studies, the final sample size became 634. A multistage sampling technique was used to select the study participants for this study. Initially, a simple random sampling technique was used to select 7 kebele from a total of 17 kebele assuming that the kebele is homogeneous. A proportional allocation of a sample was implemented for each selected kebele. From each kebele, households were selected using a systematic random sampling technique with a specified sampling interval; every 30^th^ household in each kebele was selected using lists of all households (from the existing lists of households from the registration book of health extension workers) as a sampling frame. Then, eligible individuals from the households were enrolled in the study. In cases where there was more than one eligible individual in the household, a lottery method was used to choose one among them, and if there was no eligible individual in a household, the next house was visited.

**Data collection tools and procedure:** a structured interviewer-administered questionnaire was used to collect the data. For CVD prevention practices survey designed by WHO was adapted and used [12]. The questionnaire comprised CVD preventive practices in which respondents were required to indicate, on a four-point Likert-type scale, how often they performed each preventive practice. Response options are Likert-type and range from high to low: 4-always, 3-frequently, 2-seldom, and 1-never. However, the scale was rated inversely in the case of smoking, alcohol, eating fast foods and fried foods, and salt and butter consumption. The total score ranged from 21 to 84, the higher the score, the better the CVD prevention practices would be. Cronbach´s alpha obtained for this section was 0.79. This section has six dimensions (smoking, alcohol, physical activity, diet, stress management, and health maintenance check-ups).

In addition, a cover page containing closed-ended questions collecting demographic data of the respondents was included such as age, gender, education, marital status, occupation, and income [12]. Additionally, participants were asked about personal, interpersonal, and community environment-related factors by using tools adapted from various relevant studies [13-21] The questionnaire was translated into local languages (Afaan Oromo and Amharic) by a person who is fluent in English, Afaan Oromo, and Amharic languages and was checked for consistency by a third independent person and expert in all the mentioned languages. Then, Amharic and Afaan Oromo version questionnaires were used for data collection.

Data were collected over one month by three BSc nurses who had previous data collection experience and were supervised by one MSc nurse. Closed houses were visited three times during the time of data collection while proceeding to the next house in the interval. Training was provided for the data collectors and supervisor for two days on the technique of data collection, the purpose of the study, and the content of the questionnaires by the project members. To assure the quality of data validated tool was used. Pretesting of the data collection tools was conducted at Agaro Town using 5% (32 adults) of the total sample and then the tool was improved in terms of its clarity and simplicity in collecting the data required for the study. Data collectors and supervisors check for completeness of the data every day during data collection.

### Variables and measurement

**Dependent variables:** CVD Prevention practice.

**Independent variables:** age, gender, educational level, income, occupation, marital status knowledge of CVD, attitude, self-perceived estimate of CVD risk, family and personal history of CVD, having a friend or knowing someone with CVD, self-efficacy, social support (family, friend), body weight norm, peer pressure, availability and accessibility of healthy diets, place for physical activity, working hours.

### Operational definition and definition of terms

**Cardiovascular disease (CVD) prevention practices:** are those measures undertaken to prevent the risk of developing CVDs. These include physical activity, not starting or cessation of smoking, following healthy diets, maintenance of optimal blood pressure, normal cholesterol and glucose levels, and stress management [22]. The mean value of the preventive practice questions was used to classify the respondents' preventive practices as either good or poor. Those who scored above or equal to the mean value of the preventive practice questions had good CVD prevention practices, while those who scored below the mean value of the preventive practice questions had poor CVD prevention practices [23].

**Knowledge of CVD risk factors:** categorized based on the mean score of knowledge questions either as good (greater equal to mean score) or poor (less than mean score) knowledge of CVD risk factors [23,24].

**Self-efficacy:** dichotomized based on the mean value as either high (equal to or above the mean value) or low (less than the mean value) [25,26].

**Social support:** the mean value of social support questions was used to dichotomize either adequate social support (above and equal to the mean value) or inadequate social support (below the mean value) [27-29].

**Self-perceived estimate of CVD risk:** it was assessed on a 5-point Likert-type scale and collapsed into three categories: low (1-2), intermediate (3), and high (4-5) [30,31].

**Working hours:** refers to the number of hours that a person usually works in a typical day or week, regardless of whether he/she is paid or not. Dichotomized into two (< or = 8 hours or > 8 hours) based on the normal working hours of our country´s standard (8 hours per day or 48 hours per week) [32,33].

**Statistical analysis:** following the data collection, data were rechecked for completeness and were entered into Epidata version 4.6 and then exported to SPSS version 23.0 for analysis. Appropriate coding and re-coding were done at each step for the variables as necessary. Descriptive statistics like frequencies, percentages, means and standard deviations were performed. CVD preventive practices were dichotomized into either good or poor practice based on the mean value of practice questions. Accordingly, a score equal to or above the mean value represented a good practice and a score below the mean value represented poor practice. A binary logistic regression analysis was done to sort variables candidate for multiple logistic regression having a p-value less than or equal to 0.25. Multivariable logistic regression analysis was conducted to identify factors strongly associated with cardiovascular disease prevention practice among adults. Finally, the association was declared with a p-value less than 0.05 with an adjusted odds ratio (AOR) at a 95% confidence interval. The dependent variable was checked for normality and independent variables were checked for multicollinearity. The Shaipro Wilk test results of dependent variables satisfied the assumption of normality (p = 0.064). Hosmer and Lemeshow´s test was used to determine the model fitness. Finally, the result of the analysis was presented in texts, tables, and graphs as appropriate.

**Ethics approval and consent to participate:** ethical clearance to conduct the study was obtained from the institutional review board of Jimma University, Institute of Health [IHRPG1/370/21] and submitted to each kebele administration for permission and cooperation before data collection. Verbal consent was obtained from each participant that participation was voluntary, and they had the right to withdraw at any time from the study. The consent consisted of the study purpose and procedures, potential risks and benefits, voluntary participation, and right of withdrawal; the information provided by each respondent was kept strictly confidential. Respondents were also informed that their answers to the questions would be grouped with others.

## Results

**Description of the participants:** out of 634 study participants who were identified for the study, 605 of them were included in the study with a response rate of 95.4% (Table 1). More than half (57.4%) of the respondents were females.

**Table 1:** socio-demographic characteristics of respondents among adults in Jimma Town, Southwest Ethiopia (September 2021)

Characteristics	Category	Frequency	Percent (%)
Age	18-24	164	27.1
	25-34	202	33.4
	35-44	107	17.7
	45-54	58	9.6
	55-64	38	6.3
	65 and above	36	6.0
Ethnicity	Oromo	264	43.6
	Amhara	140	23.1
	Tigre	88	14.5
	Dawuro	96	15.9
	Others£	17	2.8
Religion	Muslim	283	46.8
	Orthodox	219	36.2
	Protestant	91	15.0
	Others@	12	2.0
Educational level attended	No formal education	57	9.4
	Primary school (1-8)	231	38.2
	Secondary school (9-12)	173	28.6
	College and/above	144	23.8
Occupation	Housewife	141	23.3
	Merchant	117	19.3
	Government	114	18.8
	Non-government	100	16.5
	Self-employed	79	13.1
	Unemployed	35	5.8
	Others$	19	3.1

£: Yem, Kafa, Gurage, silte; @: Aventisit, catholic, Hawariyat, Wakefata; $: retired, student

### Personal related factors

**Figure 1:**
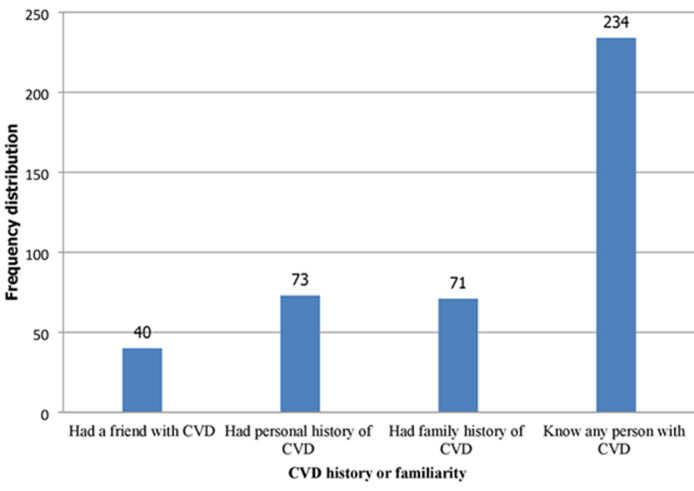
frequency distribution of participants by personal and family history status of cardiovascular disease (CVD) and familiarity with a person who had CVD among adults in Jimma Town, Southwest Ethiopia, 2021

**Having the disease (personal, family, friend, or others):** seventy-three (12.1%) of the participants had a history of CVD, 71 (11.7%) had a family history of CVD, and 234 (38.7%) knew any person with CVD condition (Figure 1).

**Knowledge of CVD risk factors:** the study showed that the mean (SD) knowledge score of CVDs risk factors among the participants was 7.01 (2.1) (Table 2).

**Table 2:** knowledge of cardiovascular disease risk factors among adults in Jimma Town, Southwest Ethiopia (September 2021)

S.N	Items	Frequency (%)	
		Yes	No
1	Do you think that smoking may increase someone’s risk of cardiovascular diseases?	519(85.8)	86(14.2)
2	Do you think that alcohol consumption can increase the risk of cardiovascular diseases?	514(85)	91(15)
3	Do you think that an unhealthy diet can increase the risk of CVD?	517(85.5)	88(14.5)
4	Do you think that physical inactivity can increase the risk of developing cardiovascular diseases?	478(79.0)	127(21.0)
5	Do you think that hypertension can increase the risk of developing cardiovascular diseases?	385(63.6)	220(36.4)
6	Do you think that diabetes can increase the risk of developing cardiovascular diseases?	345(57.0)	260(43.0)
7	Do you think that dyslipidemia can increase the risk of developing cardiovascular diseases?	468(77.4)	137(22.6)
8	Being overweight increases a person’s risk for heart disease	467(77.2)	138(22.8)
9	Do you think having a family history of cardiovascular disease increases the risk of getting CVDs	176(29.1)	429(70.9)
10	The older a person is, the greater their risk of having heart disease	373(61.7)	232(38.3)

**Attitude towards CVD prevention practice:** in this study, the mean (SD) score of respondents' attitude towards CVD prevention practice was 33.92 (4.38), with minimum and maximum scores of 16 to 40 respectively (Table 3).

**Table 3:** attitude towards cardiovascular disease prevention among adults in Jimma Town, Southwest Ethiopia (September 2021)

S.N	Items	SA (%)	A (%)	N (%)	D (%)	SD (%)
1	I should do exercise to maintain a healthy life>	290(47.9)	189(31.2)	29(4.8)	91(15.0)	6(1.0)
2	Smoking is bad for cardiovascular health	460(76.0)	84(13.9)	9(1.5)	51(8.4)	1(0.2)
3	I should control my body weight	239(39.5)	263(43.5)	38(6.3)	60(9.9)	5(0.8)
4	I should eat less fatty food for a healthy life>	218(36.0)	244(40.3)	48(7.9)	85(14.0)	10(1.7)
5	I choose not to eat or buy fast food when going out with friends	222(36.7)	220(36.4)	48(7.9)	100(16.5)	15(2.5)
6	I should avoid drinking too much sugary drinks	259(42.8)	243(40.2)	46(7.6)	54(8.9)	3(0.5)
7	I should include fruit or vegetables in my diet for maintaining my health	383(63.3)	139(23.0)	45(7.4)	33(5.5)	5(0.8)
8	I should control my stress to avoid getting CVD disease	484(80.0)	92(15.2)	12(2.0)	15(2.5)	2(0.3)

S.N: serial number; CVD: cardiovascular disease; SA: strongly agree; A: agree; N: neutral; D: disagree; SD: strongly disagree

**Participants´ self-efficacy:** in this study, self-efficacy was measured using a general self-efficacy tool. The minimum and maximum scores were 12 and 36 respectively, with a mean ± (SD) score of 26.9 ± 4.53. Based on this mean value, 341 (56.4%) of study participants had high self-efficacy.

**Figure 2:**
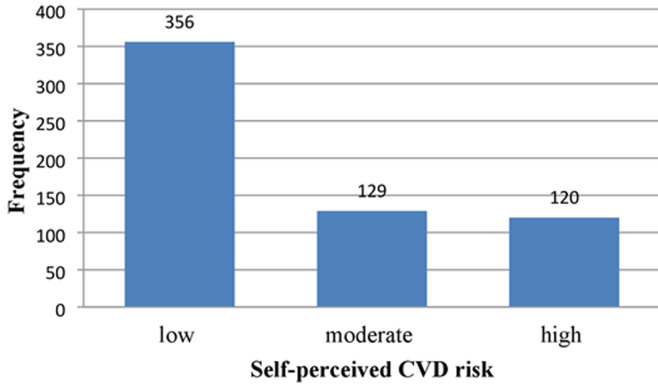
frequency distribution of self-perceived estimate of cardiovascular disease (CVD) risk among adults in Jimma Town, Southwest Ethiopia, 2021

**Self-perceived estimate of CVD risk:** regarding the self-perceived estimate of CVD risk among adults in Jimma town, 356 (58.8%) perceived they were at low risk for developing CVD in the next 10 years. Only, 120 (19.8%) perceived that they were at high risk for developing CVD in the next 10 years (Figure 2).

**Interpersonal-related factors (body weight norm, social support, and peer pressure):** with regards to body weight norm, almost more than half (53.2%) of participants reported having a partner whose body weight is higher than what is considered normal as compared to their height (overweight/obese). One hundred sixty-six (27.4%) and 150 (24.8%) of the respondents reported that the BMI (the figures) which represents overweight would be considered as normal (preferred) body weight for males and females respectively according to the expectation of people living around them.

Concerning the social support of study participants, the most frequent (29.8%) obtained social support (family, friends) item compared to others was to be encouraged not to eat unhealthy foods when they were interested in doing so. Regarding the peer pressure of adults, two hundred seventy-four (45.3%) respondents strongly agreed that they would resist pressure from their friends to drink alcohol when they were at home.

**Community environment-related factors:** the study revealed less than half (47.6%) of participants agreed that the cost of a healthy diet was affordable. However, more than three-fourths (76.9%) agreed that they had easy access to a healthy diet in a nearby home. Three hundred and twenty-three (53.4%) of participants reported that it was difficult to get a facility for physical activity near home. More than two-thirds (68.3%) of the respondents spent 8 hours or less on work every day.

**Cardiovascular disease prevention practices:** the mean (SD) score of CVD prevention practice was 48.38 ± 5.06 out of 84, with the minimum and maximum scores as 33.00 and 62.00, respectively. Accordingly, about 46.8% (95 % CI 42.8, 50.8) of the study participants had good CVD prevention practices (Table 4).

**Table 4:** cardiovascular disease prevention practices among adults in Jimma Town, Southwest Ethiopia (September 2021)

S.N	Statement	Always N (%)	Frequently N (%)	Seldom N (%)	Never N (%)
1	How often do you smoke	41(6.8)	17 (2.8)	35 (5.8)	512 (84.6)
2	How often do you have at least 6 standard drinks on one occasion	18 (3.0)	26 (4.3)	83 (13.7)	478 (79.0)
3	How often do you have at least one standard alcoholic drink	34 (5.6)	91(15.0)	142 (23.5)	338 (55.9)
4	How often do you consume any homebrewed alcoholic drink	27 (4.5)	98 (16.2)	139 (23.0)	341(56.4)
5	How often does your daily activity involve vigorous intensity activity for at least 10 minutes continuously	60 (9.9)	87(14.4)	142(23.5)	316 (52.2)
6	How often does your daily activity involve moderate-intensity activity for at least 10 minutes	111(18.3)	264 (43.6)	136 (22.5)	94(15.5)
7	How often do you do vigorous-intensity exercise for at least 10	27(4.5)	73(12.1)	131(21.7)	374(61.8)
8	How often do you do moderate-intensity exercise for at least 10	14(2.3)	54(8.9)	168(27.8)	369(61.0)
9	How often do you spend your leisure time to exercise at least 20	28(4.6)	51(8.4)	116(19.2)	410(67.8)
10	How often do you walk for at least 10 minutes from and to home per day	399(66.0)	123(20.3)	54(8.9)	29(4.8)
11	How often do you take at least 4-5 servings of fruits in your diet per day	8(1.3)	18(3.0)	159(26.3)	420(69.4)
12	How often do you take at least 4-5 servings of vegetables in your diet per day?	4(0.7)	48(7.9)	184(30.4)	369(61.0)
13	How often do you add salt or a spice-containing salt to your food right before you eat it?	341(56.4)	126(20.8)	106(17.5)	32(5.3)
14	How often do you eat fast food?	41(6.8)	114(18.8)	293(48.4)	157(26.0)
15	How often do you eat fried food?	103(17.0)	198(32.7)	217(35.9)	87(14.4)
16	How often do you use butter for food preparation?	198(32.7)	179(29.6)	186(30.7)	42(6.9)
17	How often do you manage your stress through meditation, music, movies, and travel?	227(37.5)	156(25.8)	58(9.6)	164(27.1)
18	How often do you check your body weight and compare it with your normal range of body weight	45(7.4)	155(25.6)	299(49.4)	106(17.5)
19	How often do you measure your BP at least every 2 years?	87(14.4)	119(19.7)	171(28.3)	228(37.7)
20	How often do you measure your BGL at least every 3 years?	21(3.5)	46(7.6)	128(21.2)	410(67.8)
21	How often do you measure your blood lipid profile at least every 3-4 years?	6(1.0)	15(2.5)	79(13.1)	505(83.5)

BP: blood pressure; BGL: blood glucose level

**Factors associated with cardiovascular disease prevention practices:** the bivariate analysis revealed that at p-value less or equal to 0.25 significance level 13 (occupation, educational status, marital status, personal history of CVD, knowledge, attitude, self-efficacy, self-perceived estimate of CVD risk, social support, body weight norm, accessibility of healthy diet, place for physical activity and working hours) variables were identified as; candidate variables for multivariable logistic analysis. All candidate variables were entered together into a multivariable logistic regression using the backward method to determine final predictors of CVD prevention practices by controlling for potential confounders.

In a multivariable logistic regression, five variables were found to be the statistically significant predictors of CVD prevention practices with a p-value of < 0.05 and 95% CI. Accordingly; hours spent on work per day, knowledge of CVD risk factors, self-efficacy, social support, and self-perceived estimate of CVD risk were the final independent predictors of CVD prevention practices. The overall model adequately fits the data (p = 0.674) (Table 5).

**Table 5:** multivariable logistic regression showing independent factors of cardiovascular disease (CVD) prevention practices among adults in Jimma Town, Southwest Ethiopia (September 2021)

Variables	Category (N)	CVD prevention practice level		COR (95%CI)	AOR (95%CI)	P-value
		Good (%)	Poor (%)			
Hours spent on work per day	< or =8	216(52.3)	197(47.7)	1	1	<0.001
	>8	67(34.9)	125(65.1)	0.489(0.3, 0.7) *	0.445(0.3, 0.7) **	
Knowledge of CVD risk factor	Poor	128(38.2)	207(61.8)	1	1	<0.001
	Good	155(57.4)	115(42.6)	2.180(1.57, 2-3.0) *	2.013(1.4, 2.9) **	
Self-efficacy	Low	99(37.5)	165(62.5)	1	1	0. 007
	High	184(54.0)	157(46.0)	1.953 (1.4, 2.7) *	1.670(1.1, 2.4) **	
Self-perceived estimate of CVD risk	Low	143(40.2)	213(59.8)	1	1	0.001
	Intermediate	74(57.4)	55(42.6)	2.004(1.3, 3.0) *	2.035(1.3, 3.2) **	0.003
	High	66(55.0)	54(45.0)	1.821(1.2, 2.8) *	2.101(1.3, 3.4) **	0.003
Social support	Inadequate	100(35.1)	185(64.9)	1	1	<0.001
	Adequate	183(57.2)	137(42.8)	2.471(1.8-3.4) *	2.063(1.4, 2.9) **	

* :(P<0.25) in bivariate; 1: reference group; **: statically significant in multivariate

## Discussion

This study revealed that the overall percentage of individuals who had a good CVD prevention practice was suboptimal. That means only less than half of the participants had good CVD prevention practices. Knowledge of cardiovascular disease risk factors, self-efficacy, self-perceived estimate of cardiovascular disease risk factors, social support, and working hours were found to be significantly associated with CVD prevention practice.

This finding is almost consistent with studies conducted in Nepal (48.2%) and India (49.5%) of participants had satisfactory CVD prevention practices [34,35]. This similarity might be due to the relatedness of the socioeconomic status of participants; in all studies, participants were from developing countries as well as it could also be due to sample size consistency.

On the other hand, this result is much higher than studies conducted in Uttar Pradesh in India (12.40%) and Cameroon (15%) [36,37]. The discrepancy might be due to: the small sample size and inclusion criteria in the case of the study done in Uttar Pradesh where only adults aged 20 to 40 years were included; during a period early in life adults tend to be involved in health risk behaviors like smoking, heavy drinking, unhealthy diet and less attentive to medical check-up [38]. The study done in Cameroon used items as a basis for categorizing CVD prevention practice in which a good practice was considered as having practiced five or more CVD prevention practice items, this might explain the reason for variation with the present study.

However, the findings of the study are lower than the findings of previous studies done in Japan (56.9%), Iran (63.3%), Thailand (64.90%), and Malaysia (65%) [39-42]. The discrepancy of this finding might be due to: variations in socioeconomic status and Socio-demographic background of participants. The difference in socioeconomic status between the developing country, Ethiopia, and the developed country in the case of Japan, which influences individuals' lifestyle behavior may contribute to this variation. The previous study done in Iran included adults who had previous military experience and their spouses, which may promote the development and maintenance of healthy lifestyle behaviors [43].

In the case of the study done in Thailand, the study was conducted among adults living in rural areas who are at risk for developing CVD; being rural dwellers might increase the chance of being physically active and having access to fresh fruit and vegetables rather than processed foods [4]. Additionally, knowing one's risk for developing CVD might also be accompanied by improvement in undertaking preventive measures. The possible reason for the discrepancy with the study conducted in Malaysia might also be due to the socio-demographic background of participants in which most of the participants attended tertiary education, this might lead to having more information on CVD preventive measures.

In this study, respondents who had good knowledge of CVD risk factors were two times more likely to have good CVD prevention practices. This finding is consistent with a finding of a study conducted in Nepal [34]. The possible explanation might be due to knowledge about CVD and its modifiable risk factor is a fundamental pre-requisite to change the individuals´ behaviors and lifestyle practices. It may also help reduce the individuals´ exposure to those risk factors [5, 44,45].

Similarly, this study revealed that adults who had good self-efficacy were approximately two times more likely to be involved in CVD prevention practices than those who had poor self-efficacy. It is supported by a study done in Samutprakan, Thailand [46]. This might be because self-efficacy encourages a person to take an active role in self-management activities and adopt a healthy lifestyle [47]. Therefore, the more confident individuals are the more they will be motivated to modify their lifestyle as required.

In addition, this study showed that adults who perceived that they were at intermediate and high risk of developing CVD were approximately two times more likely to have a good CVD prevention practice than those who had a low self-perceived estimate of CVD risk. This finding is consistent with the study conducted in Thailand [48]. The possible explanation might be that being aware of one's risk for developing CVD may lead to undertaking preventive measures.

This result also revealed that adults who had adequate social were 2.4 times more likely to have a good CVD prevention practice. This result is comparable with the studies conducted in the USA and Korea [14,28]. The reason might be that social support is one of the effective factors in the maintenance, continuity, and promotion of healthy lifestyle practices [49]. Those who get social support from family or friends are more likely to value and repeat their efforts for healthy lifestyle modification.

Finally, the longer working hours of the participants were found to have a significant effect by lowering CVD preventive practices by 54.9%. This finding is in line with a finding of a previous study conducted in Korea [14]. This might be because people working more hours are more likely to have poor eating habits, do less physical activity, and lead stressful lives [50j. The strength of this study is it includes a large sample size and used valid tools. Even though the study depicted significant findings on cardiovascular disease prevention practices, it is not free of limitations. One of its limitations is being a cross-sectional study which does not show cause and effect relationships. The other limitation is a recall bias because of self-reporting. Therefore, we recommend a longitudinal study to depict more significant factors.

## Conclusion

In this study the overall cardiovascular disease prevention practice among the community was suboptimal. Hours spent on work per day, knowledge of cardiovascular disease risk factors, self-efficacy, social support, and self-perceived estimate of cardiovascular disease risk were the identified independent predictors that were significantly associated with cardiovascular disease prevention practice. Therefore, it is necessary to establish effective interventional programs aimed at promoting positive health behaviors regarding cardiovascular disease risk factors in the community.

### What is known about this topic

- Cardiovascular diseases (CVDs) are a group of disorders of the heart and blood vessels, primarily due to modifiable risk factors such as smoking, unhealthy diet, hypertension, diabetes mellitus, stress, and obesity.

### What this study adds

- This study enhances communities´ health by revealing the level and associated factors of the prevention practices, aiding healthcare providers in developing effective prevention and treatment strategies;
- This research could lead to the formulation of guidelines for CVD risk screening, public health campaigns, and evidence-based interventions, thereby enhancing understanding and prevention practices;
- Additionally, the findings of this study may inform future research on the topic and contribute to the development of evidence-based intervention.

## References

[1] World Health Organization (2020). WHO reveals leading causes of death and disability worldwide: 2000-2019.

[2] World Health Organization (2021). Cardiovascular diseases (CVDs).

[3] Benjamin EJ, Blaha MJ, Chiuve SE, Cushman M, Das SR, Deo R (2017). Heart Disease and Stroke Statistics-2017 Update: A Report From the American Heart Association. Circulation.

[4] Damasceno A (2016). Noncommunicable Disease. The Heart of Africa: Clinical Profile of an Evolving Burden of Heart Disease in Africa.

[5] De Backer G (2017). Prevention of cardiovascular disease: recent achievements and remaining challenges. Eur Soc Cardiol.

[6] Liu MB (2014). Cardiovascular diseases. Chin Med J (Engl).

[7] Amegah AK (2018). Tackling the Growing Burden of Cardiovascular Diseases in Sub-Saharan Africa. Circulation.

[8] Ojji DB, Lamont K, Ojji OI, Egenti BN, Sliwa K (2017). Primary care in the prevention, treatment and control of cardiovascular disease in sub-Saharan Africa. Cardiovasc J Afr.

[9] Ali S, Misganaw A, Worku A, Destaw Z, Negash L, Bekele A (2021). The burden of cardiovascular diseases in Ethiopia from 1990 to 2017: evidence from the Global Burden of Disease Study. Int Health.

[10] Desalegn H, Fekadu S, Deribew A (2017). Clinical assessment of cardiovascular disease associated risk factors in Jimma Town, Southwest Ethopia: A community based cross-sectional study. Ethiop Med J.

[11] Tsegaye NT, Melka GA (2022). Land Surface Temperature Detection in Relation to Land Use Land Cover Change: The Case of Jimma City and It's Surroundings, Jimma Zone, Southwest, Ethiopia.

[12] World Health Organization (2017). The WHO STEPwise approach to noncommunicable disease risk factor surveillance (STEPS). Geneva.

[13] Pahn J, Yang Y (2021). Factors Associated With Cardiovascular Disease Prevention Behavior Among Office Workers Based on an Ecological Model. SAGE Open.

[14] Ejaz S, Afzal M, Hussain M, Sarwar H, Gilani SA (2018). Knowledge, Attitude, and Practice Regarding Modifiable Risk Factors of Cardiovascular Diseases among Adults in Rural Community, Lahore. Int J Soc Sc Manage.

[15] Ibrahim MM, Rahman NA, Rahman NI, Haque M (2016). Knowledge, attitude and practice of Malaysian public university students on risk factors for cardiovascular diseases. J Appl Pharm Sci.

[16] Gillison FB, Killen V, Grey EB, Standage M, Watson D, Kremers SPJ (2022). Influence of obesity prevalence on social norms and weight control motivation: a cross-sectional comparison of the Netherlands and the UK. Psychol Health Med.

[17] Schwarzer R, Luszczynska A (2005). Perceived Self-Efficacy and Health Behavior Theories. Health (San Francisco).

[18] Rieger E, Sellbom M, Murray K, Caterson I (2018). Measuring social support for healthy eating and physical activity in obesity. Br J Health Psychol.

[19] Carbonneau E, Robitaille J, Lamarche B, Corneau L, Lemieux S (2017). Development and validation of the Perceived Food Environment Questionnaire in a French-Canadian population. Public Health Nutr.

[20] Brown BW (2017). Do Personal Factors and Interpersonal Influences Affect Commitment to a Plan of Action for Physical Activity Among African-American Women?.

[21] Yahya R, Muhamad R, Yusoff HM (2012). Association between knowledge, attitude, and practice on cardiovascular disease among women in Kelantan, Malaysia. Int J Collab Res Intern Med Public Heal.

[22] Mohammad NB, Rahman NAA, Haque M (2018). Knowledge, attitude, and practice regarding the risk of cardiovascular diseases in patients attending an outpatient clinic in Kuantan, Malaysia. J Pharm Bioallied Sci.

[23] Hamilton K, Warner LM, Schwarzer R (2017). The Role of Self-Efficacy and Friend Support on Adolescent Vigorous Physical Activity. Health Educ Behav.

[24] Mszar R, Buscher S, McCann D, Taylor HL (2021). Self-Efficacy, Perceived Barriers to Care, and Health-Promoting Behaviors Among Franco-Americans Across Cardiovascular Risk Factors: A Cross-Sectional Study. Am J Heal Promot.

[25] Samuel LJ, Dennison Himmelfarb CR, Szklo M, Seeman TE, Echeverria SE, Diez Roux AV (2015). Social engagement and chronic disease risk behaviors: the Multi-Ethnic Study of Atherosclerosis. Prev Med.

[26] Cavanagh CE, Rosman L, Chui PW, Bastian L, Brandt C, Haskell S (2020). Barriers to cardiovascular disease preventive behaviors among OEF/OIF/OND women and men veterans. Heal Psychol.

[27] Kemper KA, Sargent RG, Drane JW, Valois RF, Hussey JR (1994). Black and white females' perceptions of ideal body size and social norms. Obes Res.

[28] McClendon D (2012). Perceived susceptibility of cardiovascular disease as a moderator of relationships between perceived severity and cardiovascular health-promoting behaviors among female registered nurses. Diss Abstr Int Sect B Sci Eng.

[29] Grauman Å, Veldwijk J, James S, Hansson M, Byberg L (2021). Good general health and lack of family history influence the underestimation of cardiovascular risk: a cross-sectional study. Eur J Cardiovasc Nurs.

[30] Ministry of Manpower Summary Table: Hours Worked.

[31] Ethiopian Labour Relationships Lawyer (2023). Understanding Normal Hours of Work in Ethiopia: A Guide to Labour Proclamation No 1156/2019.

[32] Vaidya A, Aryal UR, Krettek A (2013). Cardiovascular health knowledge, attitude and practice/behaviour in an urbanising community of Nepal: a population-based cross-sectional study from Jhaukhel-Duwakot Health Demographic Surveillance Site. BMJ Open.

[33] Muthukrishnan G, Kingston C, Ravikumar A (2018). A cross-sectional study of knowledge, attitude, and practice on cardiovascular disease and its risk factors. Int J Community Med Public Heal.

[34] Dayal B, Singh N (2018). Association between knowledge, attitude, and practice on cardiovascular disease among early adults of Lucknow city. Al Ameen J Med Sci.

[35] Dzalle BD, Kouam CK, Esong MB, Belobo JT, Fozin GR, Watcho P (2021). Knowledge, Attitude, and Practices on Primary Preventive Measures of Cardiovascular Diseases: A Cross-sectional Study in Dschang. West Cameroon.

[36] AlQuaiz AM, Kazi A, Almigbal TH, AlHazmi AM, Qureshi R, AlHabeeb KM (2021). Factors Associated with an Unhealthy Lifestyle among Adults in Riyadh City, Saudi Arabia. Healthcare (Basel).

[37] Goryoda S, Nishi N, Hozawa A, Yoshita K, Arai Y, Kondo K (2018). Differences in Lifestyle Improvements With the Intention to Prevent Cardiovascular Diseases by Socioeconomic Status in a Representative Japanese Population: NIPPON DATA2010. J Epidemiol.

[38] Kazemi T, Bijari B (2014). Knowledge, attitude and performance of Birjand city veterans and their wives about cardiovascular diseases risk factors. Iran J War Public Heal.

[39] Suksatan W (2018). Factors influencing health-promoting behaviors among people at risk of hypertension in a rural community, Ubon Ratchathani Province. Dis Control J.

[40] Yasin SM, Isa MR, Ismail N, Suddin LS, Saman MSA, Ismail Z (2020). The relationship between socioeconomic status and knowledge on cardiovascular risk factors and its preventive practices among urban dwellers in Selangor, Malaysia. Malaysian J Public Heal Med.

[41] Yaffe K, Hoang TD, Byers AL, Barnes DE, Friedl KE (2014). Lifestyle and health-related risk factors and risk of cognitive aging among older veterans. Alzheimers Dement.

[42] World Heart Federation (2012). Urbanization and Cardiovascular Disease. World Hear Fed.

[43] Aminde LN, Takah N, Ngwasiri C, Noubiap JJ, Tindong M, Dzudie A (2017). Population awareness of cardiovascular disease and its risk factors in Buea, Cameroon. BMC Public Health.

[44] Workina A, Habtamu A, Diribsa T, Abebe F (2022). Knowledge of modifiable cardiovascular diseases risk factors and its primary prevention practices among diabetic patients at Jimma University Medical Centre: A cross-sectional study. PLOS Glob Public Health.

[45] Hanprasitkam K, Namjuntra R, Pakdevong N, Binhosen V, Tewapitak P, Pholphoke P (2023). Factors predicting health behaviors for cardiovascular disease prevention among Rangsit University personnel. Thai J Cardio-Thoracic Nurs.

[46] Sol BG, van der Graaf Y, van Petersen R, Visseren FL (2011). The effect of self-efficacy on cardiovascular lifestyle. Eur J Cardiovasc Nurs.

[47] Prachuablarp CW (2020). Predicting factors of preventive behaviors of coronary heart disease and stroke among menopausal women. Thai Journal of Cardio-Thoracic Nursing.

[48] Rahimi Foroushani A, Estebsari F, Mostafaei D, Eftekhar Ardebili H, Shojaeizadeh D, Dastoorpour M (2014). The effect of health promoting intervention on healthy lifestyle and social support in elders: a clinical trial study. Iran Red Crescent Med J.

[49] Berniell MI (2012). The Effects of Working Hours on Health Status and Health Behaviors.

